# A yeast model for target-primed (non-LTR) retrotransposition

**DOI:** 10.1186/1471-2164-8-263

**Published:** 2007-08-03

**Authors:** Timothy JD Goodwin, Jason N Busby, Russell TM Poulter

**Affiliations:** 1 Department of Biochemistry, University of Otago, Dunedin, New Zealand; 2 School of Biological Sciences, University of Auckland, Auckland, New Zealand

## Abstract

**Background:**

Target-primed (non-LTR) retrotransposons, such as the human L1 element, are mobile genetic elements found in many eukaryotic genomes. They are often present in large numbers and their retrotransposition can cause mutations and genomic rearrangements. Despite their importance, many aspects of their replication are not well understood.

**Results:**

We have developed a yeast model system for studying target-primed retrotransposons. This system uses the Zorro3 element from *Candida albicans*. A cloned copy of Zorro3, tagged with a retrotransposition indicator gene, retrotransposes at a high frequency when introduced into an appropriate *C. albicans *host strain. Retrotransposed copies of the tagged element exhibit similar features to the native copies, indicating that the natural retrotransposition pathway is being used. Retrotransposition is dependent on the products of the tagged element's own genes and is highly temperature-regulated. The new assay permits the analysis of the effects of specific mutations introduced into the cloned element.

**Conclusion:**

This Zorro3 retrotransposition assay system complements previously available target-primed retrotransposition assays. Due to the relative simplicity of the growth, manipulation and analysis of yeast cells, the system should advance our understanding of target-primed retrotransposition.

## Background

Retrotransposons are mobile genetic elements present, often in large numbers, in most eukaryotic genomes. They spread through the copying of RNA transcripts into DNA and the insertion of these DNAs into new sites in the host genome. Most retrotransposons can be classified into one of three classes [1]. The first of these is known as the Long Terminal Repeat (LTR) retrotransposons. The integrated forms of these elements are flanked by LTRs. These elements copy their RNA into DNA within cytoplasmic particles. The completed DNA copy is then moved to the nucleus and inserted into the host genome. Members of the second major class of retrotransposons, the tyrosine recombinase (YR) retrotransposons [1-3], contain either inverted repeats or split direct repeats. They are thought to produce circular, extrachromosomal DNA intermediates which are integrated into the host genome via the actions of an encoded tyrosine recombinase. Members of the third major class [4] lack extensive repeat sequences. These elements copy their RNA into DNA directly at the targeted insertion site, using a 3' OH exposed by nicking of the target DNA as a primer. These elements are variously referred to as non-LTR retrotransposons, poly-A retrotransposons, retroposons or LINEs (for Long Interspersed Nuclear Elements). Here, we shall refer to them as target-primed (TP) retrotransposons, following the suggestions of Curcio and Derbyshire [1].

The L1 element is the most commonly encountered TP retrotransposon in mammals [reviewed in ref. [5]]. In humans, L1 is the single most abundant feature in the genome, comprising about 17% of the sequence [6], and being responsible for at least another 11% through providing the replication machinery for processed pseudogenes [7], the abundant *Alu *elements [8] and probably also SVA elements [9]. L1 elements are also likely to have played an important part in the evolution of the structure and activity of the remainder of the genome, by providing dispersed sites of sequence similarity at which recombination can occur [10], by inserting into genes altering their structure and/or regulation [11], and by carrying flanking sequences with them during transposition (L1-mediated sequence transduction) [12-17].

An intact L1 element is about 6 kb in length and consists of two long open reading frames (ORFs) which are flanked by 5' and 3' untranslated regions (UTRs) and separated by a short interORF region [5]. ORF1 encodes a protein with nucleic acid-binding and nucleic acid-chaperone activities [18-20]. ORF2 encodes a protein with endonuclease (EN) [21] and reverse transcriptase (RT) [22] domains as well as a C-terminal cysteine-rich, zinc finger-like domain (ZF) of unknown function [23]. The 5' UTR contains an internal promoter that drives transcription of the element [24]. The activity of this promoter may be influenced by the upstream flanking sequence [25]. The 3' UTR contains a poly-adenylation signal and is followed by a poly-A tail which varies in length in different copies.

Despite the abundance and importance of TP retrotransposons, many features of their replication are as yet only poorly understood. Much of what is known is based upon biochemical analyses of the target-primed R2 element from *Bombyx mori *[26,27]. R2 inserts at a specific site within the *B. mori *ribosomal RNA gene arrays. It consists of a single long ORF flanked by 5' and 3' UTRs. The ORF encodes a protein with EN and RT activities. Following translation, the proteins associate with the mRNA and the resulting ribonucleoprotein complex then associates with the target site in the genomic DNA. Here the EN domain generates a single-stranded nick in the target site, exposing a free 3'-OH group, which is then used by the RT to prime first-strand DNA synthesis, using the R2 RNA as a template. The latter steps of retrotransposition are not well understood but presumably include the nicking of the second strand of the target site, the synthesis of second-strand DNA and the sealing of the ends. This model for retrotransposition is thought to be applicable to all TP retrotransposons [21,28]. In the case of L1 (and perhaps in most TP elements), the retrotransposon-encoded proteins have a strong tendency to mobilize the RNA that encoded them, a phenomenon known as *cis*-preference [29]. This is likely mediated by the nascent proteins remaining associated with the mRNA from which they were translated.

In efforts to further dissect the replication mechanisms of TP elements, several model systems have been developed that permit the retrotransposition of marked elements of defined sequence to be followed *in vivo*. The most widely used of these are based on human or rodent L1 elements. By studying the effects of various mutations within the L1 sequence, and by analysing the structures of numerous retrotransposed copies, much has been learned about the L1 retrotransposition process and the effect this has on the host genome [[14,21,30-37]; reviewed in ref. [38]]. Nevertheless, despite the advances resulting from these mammalian systems, some aspects of the retrotransposition process, in particular the role played by host genes, might be more easily studied in simpler systems. Assays in yeasts such as *Saccharomyces cerevisiae *or *Schizosaccharomyces pombe*, with their small genomes, well understood genetics and relative ease of generating host gene knockouts, would make valuable additions to currently available assays. Unfortunately, TP elements are not found in either *S. cerevisiae *[39] or *S. pombe *[40], so such a system has not been available to date. In addition, while the Tad1 TP retrotransposon of the widely used fungus *Neurospora crassa *has been shown to retrotranspose [41], a system permitting detailed analyses of the retrotransposition of this element has not yet been developed.

**Figure 1:**
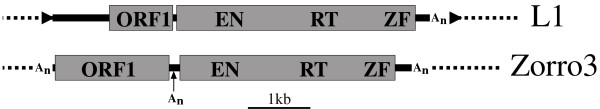
**Structures of the human L1 element and Zorro3 of *C. albicans***. Shaded boxes represent ORFs; the approximate positions of conserved protein-coding domains are indicated. The ORFs are flanked by untranslated regions (thick black lines). The border between the 3' UTR of each element and flanking genomic DNA (dashed lines) consists of a poly-A tract (An). Zorro3 also has a poly-A sequence at its 5' end and between its ORFs. The L1 element is flanked by short duplications of the target sequence (black triangles).

We recently reported the existence of three families of TP elements in the yeast *Candida albicans *[42]. We suggested that one of these, Zorro3, might make a useful yeast model for TP retrotransposition as (1) it is apparently intact (two long ORFs, the second bearing conserved EN, RT and ZF domains), (2) it is closely related to the human L1 element, (3) it is similar in structure to L1 (Fig. 1), with non-overlapping ORFs, similar protein domains, and with an ~250-bp 3' UTR terminating in a poly-A tail, and (4) it appears to have transposed recently (as suggested by variable banding patterns among different strains analysed by Southern blotting), which suggests that it has recently been active and may still be so. In this report we describe the development of an assay system for studying the retrotransposition of Zorro3. This is the first assay system for studying a TP retrotransposon in a simple microbial model system and it should significantly advance the study of these elements.

## Results

### Defining the structure of a yeast TP retrotransposon

Prior to this work only one full-length sequence of Zorro3 was available (Fig. 1), together with a single sequence of a 5'-truncated element, and two partial sequences, one containing a 5' end and the other a 3' end [42]. These were all derived from the *C. albicans *genome sequencing project strain, SC5314 [43]. Related empty sites were also available for some insertions (from SC5314 and/or other strains) allowing the 5' and 3' ends to be defined. Before attempting to develop a retrotransposition assay, we wished to obtain further data as to whether the one apparently full-length sequence was likely to represent a functional version of the element. In particular, we wished to examine the 5' end and the interORF region in more detail. The 5' end is unusual in that it begins with a poly-A sequence and in that there is only a very short distance (5 bp) between the end of the poly-A sequence and the first available ATG codon of ORF1. For the interORF region, we wished to check whether the non-overlapping ORFs and a poly-A tract found between the ORFs are general features of Zorro3 elements.

The sequences of two additional Zorro3 5' ends were obtained by inverse PCR from *C. albicans *strain ATCC10261 [GenBank:DQ239689 and GenBank:DQ239690]. Each of these elements was inserted at a different site to any Zorro3 insertion in SC5314, in agreement with previous results obtained by Southern blotting [42]. The ATCC10261 elements both have 5' ends identical to those of the two found in SC5314, i.e., beginning with a poly-A tract and with only a very short 5' UTR. In addition, comparisons between the sequences flanking these elements and the related empty sites in SC5314 (not shown), revealed that both elements had inserted into short poly-A tracts, as have the elements in SC5314 [ref. [42], and data not shown], suggesting that Zorro3 elements might preferentially integrate at such sequences.

Further sequences of the region between ORF1 and ORF2 of Zorro3 elements were obtained by PCR using DNA from ten unrelated isolates of *C. albicans*. Each additional Zorro3 sequence was found to have an interORF region identical, or very nearly so, to that of the sequenced element from SC5314, i.e., in all cases ORF1 and ORF2 do not overlap and the interORF region contains an extensive poly-A tract.

In addition, further sequences (not shown) of Zorro3-like elements were obtained from a close relative of *C. albicans*, *Candida dubliniensis*, both by PCR and by analysis of data from a *C. dubliniensis *genome sequencing project [73]. Comparisons of the elements from two different species assists in the identification of functionally important sequences, as unimportant sequences will have had time to diverge considerably. We found that the *C. dubliniensis *Zorro3-like elements have similar features to the sequenced copy of Zorro3 from *C. albicans*, i.e., they begin and end in poly-A tracts, they have only very short 5' UTRs, and ORF1 and ORF2 are non-overlapping and separated by an interORF region bearing a poly-A tract (or an extremely A-rich sequence). Furthermore, analysis of related empty sites suggested that these elements had all inserted into short poly-A tracts.

Overall, these findings indicate that the sequenced version of Zorro3 is likely to represent the intact structure of the element, despite its several unusual features.

### A retrotransposition assay for Zorro3

**Figure 2:**
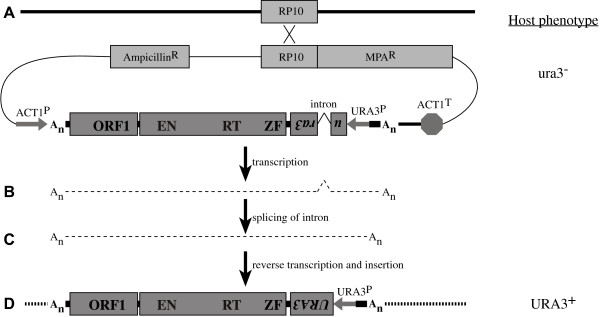
**An assay for Zorro3 retrotransposition**. (A) The cloned Zorro3 element has a retrotransposition indicator gene (*URA3 *promoter, *URA3*P, and *URA3 *ORF, disrupted by an antisense intron) inserted into its 3' UTR. The element is preceded by the *C. albicans ACT1 *promoter and followed by the *ACT1 *terminator. The transforming vector also contains sequences for selection in bacteria (Ampicillin resistance) and in *C. albicans *(MPA resistance). The entire construct can be linearised at a unique *Pin *AI site (not shown) within a copy of the *RP10 *gene to target integration to the genomic *RP10 *locus. The first step of retrotranspositon is transcription to give a full-length mRNA (B). The intron in this RNA is then removed by splicing (C). Reverse transcription and integration of the spliced RNA results in a functional and stably integrated *URA3 *gene and confers a URA3+ phenotype on the host cell (D).

Constructs for studying Zorro3 retrotransposition were built by sequentially incorporating PCR-derived sequences into bacterial plasmids as described in the Methods section. The construct used in much of the work described here, pPZ3TA (Fig. 2; GenBank accession no. EF667963), contains a full-length Zorro3 element with a retrotransposition indicator gene inserted into its 3' UTR. The retrotransposition indicator gene is the same as that used previously to study retrotransposition of the *C. albicans *LTR retrotransposon Tca2 [44], and consists of the ORF of the *C. albicans URA3 *gene and 136 bp of its promoter sequence, with the ORF disrupted by an antisense intron. This indicator gene is inserted into the Zorro3 3' UTR so that the *URA3 *ORF is on the opposite strand to the Zorro3 ORFs. The Zorro3 element in pPZ3TA is preceded by 1023 bp from the *C. albicans ACT1 *gene promoter region and is followed by 151 bp of its natural 3' context, then 391 bp from the *ACT1 *terminator region. The plasmid also contains an allele of the *C. albicans IMH3 *gene [45,46] that confers resistance to the antibiotic mycophenolic acid (MPA), for selection of the plasmid in *C. albicans*, and the ORF region of the *C. albicans RP10 *gene [47]. The plasmid can be linearised at a unique *Pin *AI site within the *RP10 *ORF to target integration to the genomic *RP10 *locus. This site has been suggested as a good site for integration and expression of transforming DNA in *C. albicans *[48].

The strategy by which plasmid pPZ3TA can be used to study retrotransposition of the cloned Zorro3 element (Fig. 2) is similar to that used previously for other systems [14,49,50]. First, the plasmid is introduced into a auxotrophic strain of *C. albicans*, CAI4 [51], which has had both copies of its *URA3 *gene deleted, using the MPA-resistance gene for selection. The transformants will initially remain auxotrophs as the marker gene on the plasmid is inactive due to the antisense intron. Splicing of the intron from a sense transcript of the marked Zorro3 element, and retrotransposition of the spliced transcript, i.e., its copying into DNA and integration into the host genome, will, however, result in the appearance of a stably integrated, functional *URA3 *gene. Expression of this gene would confer a URA3+ phenotype on the host cell. Thus, retrotransposition events can be detected by the appearance of URA3+ colonies on the appropriate selective media.

### A marked Zorro3 element retrotransposes in *C. albicans*

**Figure 3:**
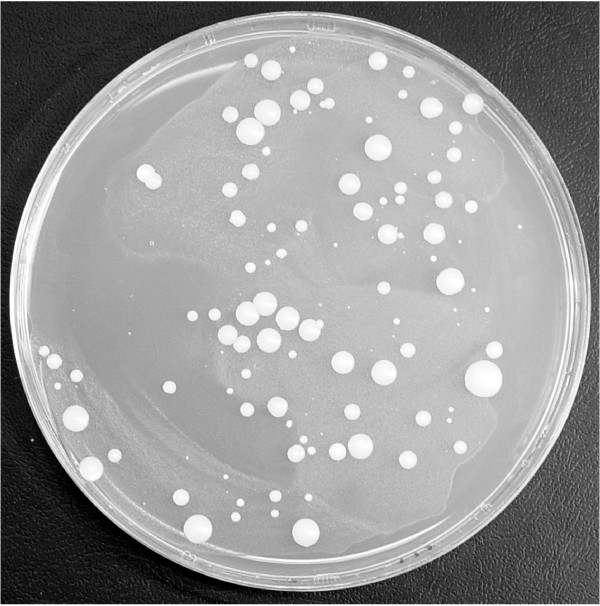
**URA3+ colonies from the Zorro3 retrotransposition assay**. An example of a retrotransposition assay plate. *C. albicans *ura3- strain CAI4, transformed with a construct containing a wild-type tagged Zorro3 element, was plated onto medium lacking uridine. The photo shows the plate after 3 weeks incubation at 22°C. No colonies were ever observed when parental (non-transformed) CAI4 was tested under identical condiions.

Plasmid pPZ3TA was introduced into *C. albicans *strain CAI4 using selection for resistance to MPA. The resulting MPA-resistant transformants were isolated, grown in liquid culture, then plated onto selective media lacking uridine and incubated at 22°, 27° or 37°C. No colonies arose on the 37° plates, but, after four to five days, colonies, representing potential retrotransposition events, began to appear on the 22° and 27° plates (Fig. 3).

To test whether these colonies result from genuine retrotransposition events (rather than from some other undefined process) the structures of their *URA3 *genes were examined. PCR amplification of DNA isolated from the URA3+ colonies using primers that flank the intron insertion site in the antisense *ura3 *gene produced two bands, one of a size expected from the introduced tagged Zorro3 element and the other slightly smaller (not shown). For two independent colonies this smaller band was cloned and sequenced. In both cases a *URA3 *gene from which the antisense intron had been precisely removed was detected. This indicates that the marker gene is derived from a spliced mRNA molecule and that the element has therefore been retrotransposed.

**Figure 4:**
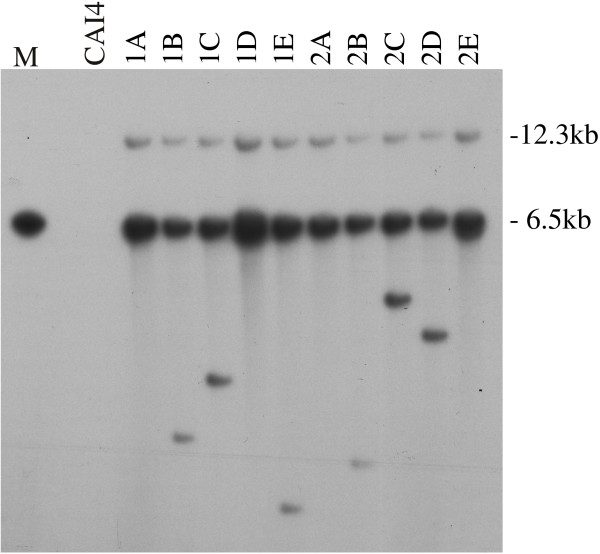
**Genomic Southern blot showing new copies of tagged Zorro3 elements in URA3+ derivatives**. The blot was probed with the *C. albicans URA3 *gene. Each lane contains DNA cut with *Hin *dIII. Lane M contains the transforming vector. CAI4 is the ura3- host strain. The 'A' lanes contain DNA from two independent ura3- transformants. Lanes B-E contain DNA from four independent URA3+ derivatives. The additional bands in these lanes represent newly retrotransposed copies of the tagged Zorro3 element. Bands at 6.5 kb (the same size as that present in the marker lane) likely represent tandem integrations of the vector in the genome. The bands at 12.3 kb are of the size expected for the sequence at the border between the tandem copies of the vector and the genomic insertion site.

Eight different URA3+ derivatives, four from each of two independent pPZ3TA transformants, were analysed by Southern blotting to determine whether the retrotransposed copies of the tagged Zorro3 element had inserted into different sites in the genome, as expected for genuine retrotransposition events (Fig. 4). This was indeed found to be the case. Each URA3+ derivative had a single additional band hybridising to a *URA3 *probe relative to their ura3- parents, and these bands were of different sizes in each of the eight strains analysed. This work suggests that the URA3+ cells derived from pPZ3TA transformants are a result of genuine retrotransposition events, and furthermore, they show that most, if not all, of these colonies have arisen independently.

### The 3' ends of retrotransposed Zorro3 elements

As a first step towards a detailed examination of the structure of the retrotransposed copies of the tagged Zorro3 elements, the 3' ends of 30 independent copies were amplified by inverse PCR and sequenced (Table 1). The 3' end was chosen for initial analysis for two reasons: one, it is close to the marker gene, so a primer straddling the insertion site of the intron in the marker can be used, which makes the reaction specific for retrotransposed copies of the element (i.e. endogenous elements and the donor marked elements, still containing the intron, are not amplified), and two, TP retrotransposons are often variably 5' truncated, leading to difficulties in designing a primer pair capable of amplifying all 5' ends.

**Table 1:** Features of retrotransposed copies of tagged Zorro3.

Insert no.	Assembly 19 contig^A^	Chromo- some	Length of target poly-A tract (bp)	5' end^B^	Length of 5' poly-A tract (bp)	3' end^C^	Length of 3' poly-A tract (bp)
12.1	x0254 (94830 F)	3	10	5' truncated (ORF2)	10	Native	20
12.2	x0257 (1660 F)	2^D^	9/11^E^	*ACT1 *promoter (52–53)	6	*URA3 *promoter	31
12.4	10119 (254650 F)	2	12	Native (recombinant)	26^F^	Native	20
22.1	x0163 (134670 R)	1	12	Native (recombinant)	20^F^	Native	20
13.3	x0234 (49865 R)	2	9	Native (recombinant)	26^F^	Native	20
13.4	10231 (18975 F)	4	10	Native	22	Native	13
13.5	x0143 (96420 F)	2	12	Native	28	3' UTR	7
13.6	x0163 (203035 F)	1	13	Native (recombinant)	20^F^	*URA3 *promoter	43
13.7	x0119 (244340 F)	2	12	Native (recombinant)	26^F^	Native	20
13.11	10063 (36770 F)	R	11	Native (recombinant)	34^F^	Native	20
13.13	x0216 (94130 R)	1	9	None (recombinant?)^G^	NA	Native	12
13.14	10212 (133650 R)	4	10	None (recombinant?)^G^	NA	Native	11
13.15	x0196 (22910 F)	2	21	*ACT1 *promoter (20–27)	20	Native	17
13.17	x0166 (27780 R)	4	12	None (recombinant)	NA	Native	25
13.18	x0163 (15740 F)	1	12	Native	~160^H^	Native	24
14.1	x0087 (72350 R)	1	10	Native	19	Native	18
14.4	20236 (26790 R)	3	7	*ACT1 *promoter (48–51)	30	Native	17
14.5	x0218 (144180 R)	1	17	Native	~100	Native	24
14.7	x0188 (25725 F)	R	9	Native	52	Native	18
14.11	x0139 (200180 F)	2	11	None (recombinant)	NA	Native	15
14.13	10020 (2820 R)	1	10	*ACT1 *promoter (20–27)	87	Native	24
14.15	x0087 (72350 R)	1	10	None (recombinant)	NA	Native	12
14.16	2500 (72900 R)	3	11	*ACT1 *promoter (20–27)	10	Native	14
14.17	x0073 (13260 R)	1	10	Native	36	Native	14
14.18	x0163 (134670 R)	1	12	Native	24	Native	25
14.19	2506 (28525 R)	7	4	Native	27	Native	16
14.20	20161 (92640 R)	R	10	Native	27	Native	16
14.21	20216 (97180 F)	1	18	Native	51	Native	17
14.22	10119 (254650 F)	2	12	*ACT1 *promoter (20–27)	25	Native	15
14.24	20155 (47610 R)	5	10	Native	37	Native	21

^A ^Assembly 19 contigs generally come in pairs of similar sequence representing portions of putative homologous chromosome pairs. Members of the pairs are distinguished by either a 1 or 2 at the start of the contig number, eg. 10254 and 20254. In the cases where the Zorro3 insertion site can be assigned to a particular homolog the number of that contig is shown. In cases where the insertion could lie on either of the homologs the first digit of the contig number is replaced by an x. The positions of the insertions within the contigs and their orientations, forward (F) or reverse (R), are shown in parentheses.

^B ^The nature of the 5' end of each element is given. For a majority of elements the 5' ends are the same as those of the full-length endogenous insertions (Native). Several elements were greater than full-length, i.e., extending back into the *ACT1 *promoter sequence. The precise lengths of *ACT1 *promoter sequences in these elements could not be determined as in all cases the similarity between the retrotransposed sequences and the *ACT1 *promoter sequences ended within runs of A residues. Some elements had recombined with endogenous full-length Zorro3 elements (Native (recombinant)). Some elements had recombined with an endogenous 5' truncated/inverted Zorro3 element in such a way that the resulting insertion lacks a 5' end (None (recombinant); see text).

^C ^The nature of the 3' end of each element is given. For most elements the 3' ends are the same as those of the endogenous insertions (Native). Some end earlier within either the Zorro3 3' UTR or within the *URA3 *promoter sequence.

^D ^Insertion site corresponds to chromosome 2 in *C. albicans *strain WO-1. The chromosomal origin of contigs 19–10257 and 19–20257 in strain SC5314 is not known due to conflicing data.

^E ^The length of the target poly-A tract varies in the two homologs.

^F ^The length of the 5' poly-A tract in each of these insertions is determined by the length of the 5' poly-A tract of the endogenous element that was involved in the recombination event.

^G ^Data not conclusive for these two insertions.

^H ^Several T residues are found within the 5' poly-A tract of this element. NA Not applicable.

We found that 27 of the 30 new retrotransposed copies of Zorro3 extended to the natural 3' end of Zorro3, one ended upstream of the natural 3' end within the Zorro3 3' UTR, and two ended even further upstream within the promoter region of the *URA3 *marker gene. None of the 30 contained any 3' transduced sequence, i.e., the 3' flanking sequence of the donor element (151 bp of its natural context followed by the *ACT1 *terminator) was not retrotransposed. All 30 copies (including those terminating upstream of the natural 3' end) ended in a poly-A tract. These varied in length between seven and 43 bp, with 21 of the 30 being between 15 and 25 bp long, inclusive.

### Insertion sites of Zorro3 elements

**Figure 5:**
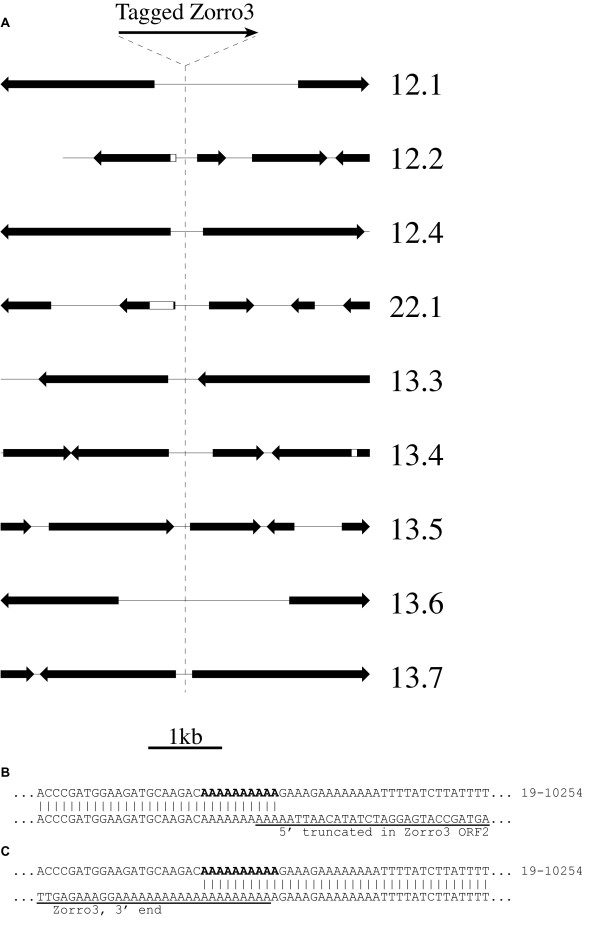
**Insertion sites of retrotransposed copies of tagged Zorro3**. (A) Genomic features in the 2.5 kb surrounding the insertion sites of tagged Zorro3 elements (dashed line) for the first nine events analysed are shown. ORFs and their 5'-3' orientations are indicated by the black arrows. Introns are indicated by white boxes within the arrows. (ORF locations were derived from the known *C. albicans *genome sequence [52]). (B and C). An example of the 5' end (B) and 3' end (C) sequence of an insertion of a tagged Zorro3 element (insertion no. 12.1). The poly-A tract at the target site is indicated in boldface. The extent of sequence matching the tagged Zorro3 element is indicated by underlining. The target site sequence in this case comes from the genome sequence, contig19-10254 (Table 1).

Sequencing of the inverse PCR products revealed not only the structure of the 3' ends of the elements but also their 3' flanking sequences. The latter could then be used to determine (by comparison with the known *C. albicans *genome sequence [52]) the nature of the insertion site prior to the retrotransposition event and the context of the insertion site in relation to other genomic features. The insertion sites were found to be distributed throughout the *C. albicans *genome, with representatives found on seven of the eight chromosomes and more events on the larger chromosomes (1, 2 and R) than on smaller ones (Table 1). Most elements were found to have inserted at unique sites, although three pairs of elements inserted at the same sites were found (insertions 12.4 and 14.22, 22.1 and 14.18, and 14.1 and 14.15). For two of these pairs the same site was certainly targeted independently as the URA3+ colonies were isolated independently from ura3- parents. Each of the new insertion sites was distinct from any previously identified Zorro3 insertion site. The new retrotransposed copies were all found to have integrated at short poly-A sequences, varying in length between 4 and 21 consecutive A residues, with 25 of 30 being between 9 and 15 bp (inclusive) in length. None of the elements had inserted into an ORF, despite coding regions comprising 61.5% of the *C. albicans *genome [53]. Many of the elements, however, had inserted very close to coding regions (Additional file 1; examples shown in Fig. 5) and there seemed to be a bias towards locations near the 5' ends of ORFs, with 16 of 30 insertions being between divergently transcribed ORFs, 13 between those in a head-to-tail arrangement and only one between ORFs that are convergently transcribed (Additional file 1). No particular association could be identified between the insertion sites and other identifiable genomic features, such as tRNA genes, subtelomeric regions, or retrotransposon LTRs and other repetitive sequences. The findings suggest that the tagged Zorro3 elements specifically integrate at poly-A sequences and may be biased towards promoter regions. This is similar to the potential preferences [outlined above and in ref. [42]] that have been identified for endogenous Zorro3 insertions.

### The 5' ends of retrotransposed Zorro3 elements

Once the 3' flanking sequence of a particular Zorro3 insertion was identified we used the *C. albicans *genome sequence data to predict what the 5' flanking sequence would be. Primers designed to this sequence were then used in PCRs in combination with a series of reverse primers along the length of Zorro3 (thus allowing for the possibility of variably 5' truncated elements) to identify the 5' end of the insertion. By this method we identified the 5' ends of 19 of the 30 insertions (Table 1). Twelve of these are full-length elements, ending precisely at the natural 5' end. Six insertions are greater than full-length, i.e., extending beyond the natural 5' end to include up to 53 bp of the 3' end of the *ACT1 *promoter sequence. Similar transductions of 5' flanking sequence have been observed for L1 elements [6,34]. The 19th insertion is 5' truncated within the ORF2 region. Each element begins with a poly-A sequence, between six and ~160 residues in length. The 5' flanking sequence of each insertion extends to the genomic poly-A tract that appears to have been targeted by the element, i.e., there are no large deletions or duplications at the target sites (Fig. 5B &5C).

The 5' ends of the remaining eleven insertions could not be identified by the above method. The 5' end of one of these insertions was, however, subsequently identified using inverse PCR and it was found to coincide precisely with the 5' end of one of the endogenous full-length Zorro3 insertions. This suggested that a recombination event had occurred between the new insertion of the tagged element and the endogenous element. We therefore designed primers to regions upstream of each of the full-length Zorro3 elements in the genome (two endogenous elements and the element on the transforming vector) and used these primers in PCRs in combination with primers specific to each of the tagged elements whose 5' ends had not yet been identified. An additional five elements (six in total) were found to have recombined with full-length endogenous insertions (Table 1). Three had recombined with an endogenous insertion on chromosome 2, one with an endogenous insertion on chromosome R and two with a full-length element on chromosome 1 that had been introduced on the transformation vector. In each case the new retrotransposed element and the endogenous full-length element that it had recombined with were located on the same chromosome. The distance between the insertion sites for elements of each pair ranged from about 40 bp to about 115 kb (not shown). Five of the six pairs of recombining elements were located in the same orientation and the recombination events presumably resulted in the deletion of the intervening sequences. Events very similar to these have been found associated with new L1 retrotransposition events in mammalian cells [32,34,35]. These chimaeric elements were proposed to arise during target-primed reverse transcription by recombination between the single-stranded L1 cDNAs produced during the initial stages of the reaction and the endogenous elements. A similar process could have given rise to the chimaeric Zorro3 insertions.

The 5' ends of the remaining five insertions could not be detected by any of the above methods. Unexpectedly, however, for several of these elements we could obtain PCR products using single primers located downstream of the 3' ends of the elements. Sequencing of these PCR products and additional PCR experiments (not shown) indicated that these elements had undergone recombinations with an endogenous Zorro3 element located on chromosome R that is 5' truncated and also includes 1.4 kb of inverted Zorro3 sequence. The recombination events had lead to the duplication of the 3' flanking sequence to each end of the now recombinant 5' truncated/inverted element and the loss of the 5' flanking sequence. None of the 3' flanking sequences of tagged insertions involved in these events were located on chromosome R. The recombination events generating these elements must have resulted from inter-chromosomal recombination and presumably generated dicentric recombinant chromosomes whose resolution would have required chromosomal breakage.

### Zorro3 retrotransposition is temperature-regulated

**Figure 6:**
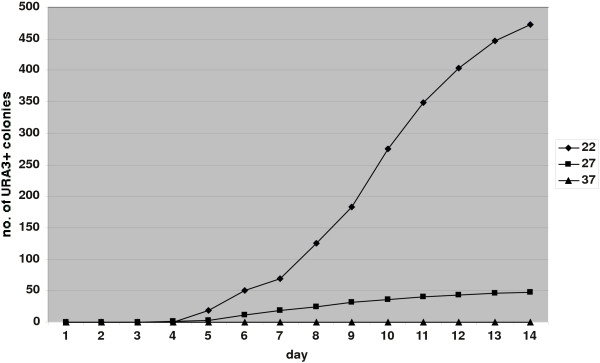
**Temperature-dependence of Zorro3 retrotransposition**. Liquid cultures of two ura3- transformants containing tagged Zorro3 elements were aliquoted onto 3 sets of plates lacking uridine. Each set of plates was incubated at 22°, 27° or 37°C for 14 days. The lines show the combined results of daily counts of URA3+ colonies for each set of plates. Identical trends were observed for each independent analysis (not shown).

As alluded to above, strains carrying the tagged Zorro3 element did not give rise to URA3+ colonies at 37°C, but produced an abundance of URA3+ derivatives at lower temperatures. To analyse this phenomenon in more detail we measured the transposition rate at 22°, 27° and 37°. We did not observe any retrotransposition at 37°, whereas numerous events were observed at 27°, and the rate was increased about a further ten-fold at 22° (Fig. 6), suggesting that some aspect of the Zorro3 retrotransposition pathway is strongly affected by temperature. It is not yet known what this is, but possibilities include some temperature-sensitive aspect of enzyme activity or perhaps the involvement of some temperature-sensitive (unstable) RNA secondary structure in the process. This fortuitous temperature-sensitivity of Zorro3 retrotransposition provides a convenient means of regulating the process, i.e., retrotransposition can be inhibited by maintaining the cells at 37°C, then allowed to proceed by moving them to a lower temperature. It is interesting to note that a temperature-dependent affect on retrotransposition was also observed for the *C. albicans *Tca2 retrotransposon [44], although in this case retrotransposition was more frequent at higher temperatures. Likewise, Ty1 of *S. cerevisiae *is temperature-sensitive, with retrotransposition events most frequent at about 24°C and very rare at 37°C [54,55].

### Zorro3 retrotransposition utilises its own proteins

**Figure 7:**
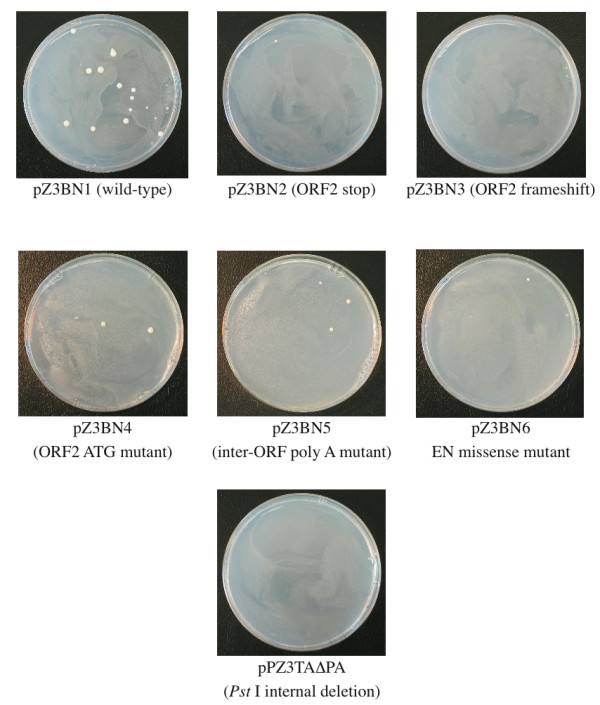
**Retrotransposition assay plates for wild-type and mutant Zorro3 elements**. Photographs are shown of representative plates inoculated with 250 μl of cultures of *C. albicans *cells transformed with wild-type or mutant Zorro3 elements and incubated at 22°C for 21 days. No colonies were ever observed when the non-transformed parent strain was tested under identical conditions.

**Figure 8:**
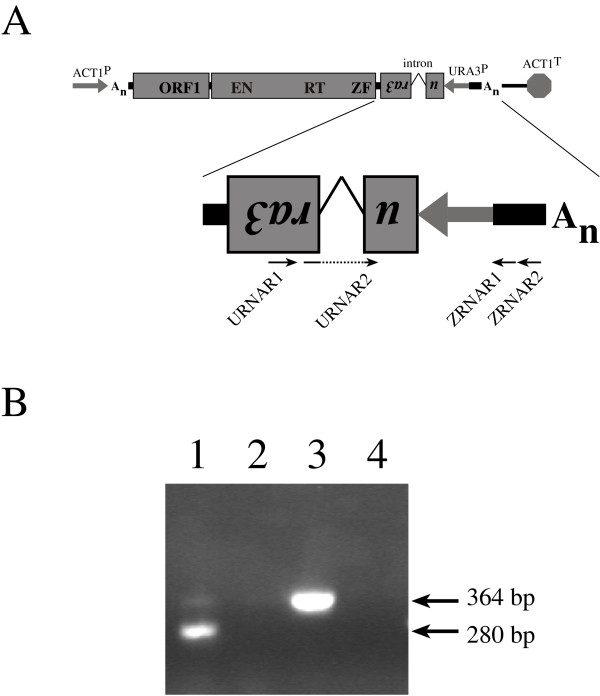
**Expression and splicing of tagged Zorro 3 RNA**. (A) Map of tagged Zorro3 element showing primer locations. URNAR2 straddles the intron insertion site. (B) RT-PCR of tagged Zorro3 RNA. Genomic DNA and total RNA were separately extracted from a *C. albicans *CAI4 derivative stably transformed with a plasmid carrying a marked Zorro3 element. The RNA was reverse transcribed using oligonucleotide ZRNAR2 as a primer. PCRs were then performed using oligonucleotides URNAR1 and ZRNAR1 as primers. Templates in the reactions were as follows: lane 1, total RNA reverse transcribed with primer ZRNAR2; lane 2, total RNA not reverse transcribed; lane 3, genomic DNA; lane 4, no template. The intron-containing PCR amplicon obtained using primers URNAR1 and ZRNAR1 would be 364 bp long. Amplification of reverse transcribed RNA from which the intron had been precisely spliced would produce an amplicon of 280 bp. Primer URNAR2 was used (in combination with ZRNAR1) to quantify the tagged Zorro3 RNA (see text).

We next asked whether Zorro3 retrotransposition is dependent on the ORFs of the cloned element or whether it is due to activation *in trans *by endogenous retroelements or cellular proteins. We made several mutant elements carrying specific alterations in the Zorro3 sequence. One mutant, pPZ3TAΔPA, has an extensive deletion that extends from a *Pst *I site at position 1337 of the Zorro3 sequence to a *Pst *I site at position 3724 and removes the last 531 bp of ORF1, the interORF region and the first 1682 bp of ORF2. A second mutant, pZ3BN2, has an inframe stop codon introduced near the start of ORF2, downstream of the first conserved domains of the EN-coding sequence. A third mutant, pZ3BN3, has a frameshift introduced into ORF2 at the same site as the stop codon in pZ3BN2 above. These mutant elements were transformed into *C. albicans *and the associated retrotransposition frequency was measured and compared with that of wild-type elements (Fig. 7, Table 2). Cells carrying pPZ3TAΔPA did not give rise to any URA3+ cells. Cells carrying pZ3BN2 or pZ3BN3 did produce URA3+ derivatives but at less than 5% the frequency of cells carrying wild-type elements. These results suggest that retrotransposition of a Zorro3 element, for the most part, requires its ORFs to be intact. One potential alternative explanation, however, is that the mutations in the ORFs of these elements result in less stable RNA molecules, and it is reduced RNA levels, rather than an absence of appropriate proteins, that results in the reduced retrotransposition frequency. To test this we measured the Zorro3 RNA levels in cells carrying wild-type and mutant elements (Fig. 8, Table 3). Cells carrying pZ3BN2 or pZ3BN3 were found to have, on average, higher Zorro3 RNA levels than cells with the wild-type element. The RNA levels in cells with the internally deleted element were measured as somewhat lower than those of cells with the wild-type element, but this slight reduction is unlikely to account for the complete absence of retrotransposition in these cells. Overall, these results show that most (> 95%) of the retrotransposition that is observed for the tagged wild-type elements in this system is dependent upon the proteins produced by the tagged element. A low background rate of retrotransposition associated with some mutants might be due to the mutations being slightly leaky or a result of *trans*-activation by endogenous elements (see the Discussion).

**Table 2:** Retrotransposition frequencies of wild-type and mutated tagged Zorro3 elements.

Transforming plasmid	Mutation	Retrotransposition frequency (URA3+ colonies/10^6 ^cells)^A, B^	Relative retrotransposition frequency (% of wild-type)	No. of assays^C^	Individual retrotransposition frequencies
pZ3BN1	Wild-type	4.47	100	10 (8)	0.664, 0.882, 1.130, 1.631, 1.641, 1.783, 2.344, 2.989, 15.339, 16.368
pZ3BN2	ORF2 stop	0.20	4.4	8 (6)	0.037, 0.043, 0.051, 0.129, 0.148, 0.170, 0.211, 0.795
pZ3BN3	ORF2 frameshift	0.16	3.6	5 (5)	0.000, 0.033, 0.053, 0.250, 0.469
pZ3BN4	ORF2 ATG → ACG	0.20	4.5	4 (4)	0.021, 0.107, 0.333, 0.353
pZ3BN5	InterORF poly-A deletion	2.20	49.2	9 (7)	0.110, 0.262, 0.263, 0.290, 0.329, 0.345, 0.608, 1.875, 15.739
pZ3BN6	EN missense	0.09	1.9	5 (5)	0.000, 0.015, 0.065, 0.093, 0.257
pPZ3TAΔPA	Internal deletion	0.00	0.0	8 (6)	0.000, 0.000, 0.000, 0.000, 0.000, 0.000, 0.000, 0.000,

^A ^The numbers of URA3^+ ^colonies were counted after 21 days incubation at 22°C.

^B ^No URA3^+ ^colonies were ever observed with the parental strain, CAI4, under identical conditions.

^C ^The number of independent transformants analysed is shown in parentheses.

### Analysis of specific mutants of Zorro3

One of the main potential uses for a yeast TP retrotransposition assay is the study of the contribution of specific sequences to the retrotransposition process. To begin an analysis of the sequences important for Zorro3 retrotransposition we made three more mutant elements. In the first additional mutant, pZ3BN4, the first ATG codon in ORF2 was changed to ACG. This triplet is of particular interest as the means by which ORF2 is translated in L1 and L1-like elements is not well understood. Because ORF2 is the second ORF in a dicistronic transcript, its translation is unlikely to be initiated simply following cap-recognition and scanning. In the next mutant, pZ3BN5, an extremely A-rich sequence in the interORF region (TA4CA5CTGATA5CACTAGA19GAGA3GACA2CA3) was replaced with an *Asp *718I site (GGTACC). In the third, pZ3BN6, a missense mutation (N17A) was made in ORF2 converting a highly conserved Asn codon in the endonuclease coding region to an Ala codon. A similar mutation at the homologous position of the human L1 element (N14A) apparently eliminates L1 EN activity and reduces retrotransposition frequency to 1% of wild-type [21].

The mutant elements were transformed into *C. albicans *and the retrotransposition frequency associated with each measured and compared to that of wild-type elements (Table 2). The levels of Zorro3 RNA for each were also measured and were all found to be similar to cells bearing the wild-type construct (Table 3).

**Table 3:** Comparisons of relative retrotransposition frequencies and RNA levels for Zorro3 mutants

Transforming plasmid	Mutation	Relative retrotransposition frequency (% of wild-type)^A^	Relative Zorro3 RNA levels (% of wild-type)^B^
pZ3BN1	Wild-type	100	100
pZ3BN2	ORF2 stop	8	288
pZ3BN3	ORF2 frameshift	1	209
pZ3BN4	ORF2 ATG → ACG	18	130
pZ3BN5	InterORF poly-A deletion	15	98
pZ3BN6	EN missense	5	55
pPZ3TAΔPA	Internal deletion	0	34

^A ^The relative retrotransposition frequency was calculated in the same way as for Table 2, but data was taken from only the particular transformants in which RNA levels were also measured.

^B ^Zorro3 RNA levels were measured as described in the text. The Zorro3 RNA levels in each RNA sample were then normalized against the ACT1 RNA levels in the same RNA sample and averaged for each set of transformants containing the same plasmid construct.

For cells carrying pZ3BN4, the retrotransposition frequency was reduced to less than 5% of that of cells bearing wild-type elements, a similar level to that found with elements bearing stop codons or frameshifts within ORF2. This indicates that the first ATG codon in ORF2 is important for Zorro3 retrotransposition (see the Discussion).

The deletion of the A-rich sequence in the interORF region (pZ3BN5) reduces the retrotransposition frequency to about half that of wild-type elements (Table 2), suggesting that this sequence is also involved in some way in retrotransposition, but that it is perhaps not as critical as the structure of ORF2.

Like the ORF2 nonsense, frameshift and ATG mutations, the N17A missense mutation in the EN coding region of ORF2 (pZ3BN6) reduces the retrotransposition frequency to less than 5% of that of the wild-type element. This suggests that the Zorro3 endonuclease is involved in the majority of the observed retrotransposition events.

## Discussion

Here we have described a system for studying the lifecycle of a TP retrotransposon in a yeast. This is the first such system in a experimentally tractable microorganism. Retrotransposed copies of the marked Zorro3 element that the system uses resemble endogenous insertions suggesting that the natural retrotransposition pathway is being employed. Retrotransposition is dependent on the element's own internal sequence, as the introduction of debilitating mutations greatly reduces the rate of, or eliminates, retrotransposition. Retrotransposition occurs sufficiently frequently to allow the process to be studied by comparing the retrotransposition frequencies of wild-type elements and elements carrying specific mutations. This yeast system complements other currently available systems for studying TP retrotransposons, which involve the analysis of tagged elements in mammalian cells [14,21,29,30,33,36,37]. For instance, it is simpler to grow and experimentally manipulate yeast cells than cultured mammalian cells. Certain experiments can be performed in yeast, such as comparing retrotransposition frequencies in multiple strains with different host genes deleted, that would currently be impractical, if not impossible, to perform in mammalian cells. In addition, the acquisition and interpretation of experimental data should be simpler in a yeast system due to the small size of yeast genomes, the low number of endogenous retrotransposon copies and the possibility than even this small number of endogenous insertions could be specifically deleted. While certain features of the L1 system and other TP retrotransposons will only be revealed by experiments performed in their natural hosts, this Zorro3 assay should make a very useful addition to the currently available systems for studying general features of TP retrotransposition.

The use of this assay system has already uncovered some interesting aspects of Zorro3 retrotransposition. For instance, we found that Zorro3 appears to always integrate at poly-A sequences. This preference may represent an adaptation to provide a large number of potential insertion sites, as such sequences appear to be much more common in the *C. albicans *genome than would be expected by chance: in a random sample of Assembly 19 contigs containing 595,932 bp of genomic DNA (4% of the 15 Mb genome) we identified 299 poly-A or poly-T (i.e., poly-A on the complementary strand) sequences 10 or more bases in length. This 596 kb random sample of the genome contained 66.7% A or T residues, so the number of 10-bp poly-A or poly-T stretches expected by chance can be estimated as about 20 (i.e., 0.334^10 ^× 595932 × 2). Poly-A sequences 10 bases in length thus appear about 15 times more frequently than expected. Assuming that the sample analysed is representative of the genome, we estimate that there are about 7500 poly-A stretches 10 or more bases long in the haploid *C. albicans *genome. Given this number of potential Zorro3 insertion sites it is interesting that in our sample of 30 new insertions we identified three pairs of elements that had independently inserted at the same sites. This finding suggests that additional factors may be involved in target site selection for Zorro3, for example, proximity to RNA polymerase II promoter regions (see below).

Targeting of poly-A sequences may also be an integral part of the retrotransposition process for Zorro3: for minus strand synthesis, the Zorro3 endonuclease would cleave the strand complementary to the target poly-A sequence, i.e., a poly-T sequence. This poly-T 'flap' could then anneal to the poly-A tract at the 3' end of the Zorro3 mRNA and the 3' OH group of the T residue adjacent to the nick could be used as a primer for minus strand DNA synthesis. For plus strand DNA synthesis, the target poly-A tract exposed by synthesis of minus-strand DNA onto the target's complementary poly-T tract, would anneal to the poly-T tract at the 3' end of the Zorro3 minus-strand DNA (corresponding to the poly-A tract at the 5' end of the mRNA) and a 3' OH group exposed by nicking within the target poly-A sequence could be used to prime plus-strand DNA synthesis. Thus, annealing between sequences at the target and the 5' and 3' ends of the Zorro3 element could assist in directing the TP reverse transcription reactions to the correct 5' and 3' ends, and thus promote the formation of full-length (potentially retrotransposition-competent) progeny elements. Interestingly, the apparent target preference for Zorro3 is similar to that for L1 [56], which preferentially integrates at runs of A residues downstream of T residues (TT/AAAA, where/represents the insertion site of the 3' end). Zorro3 and L1 elements may have a similar mechanism for attaching the 3' end of the transcript to the genomic target to initiate the TP reverse transcription reaction.

We also observed a bias against Zorro3 insertions within ORFs (with none of the 30 new insertions analysed here lying within an ORF, despite ORFs comprising more than half the genome) and an apparent bias for Zorro3 insertions a short distance (< 1000 bp) upstream of ORFs, with 17 out of 30 insertions being within 500 bp of the start of an ORF and 23 out of 30 within 1000 bp. This may simply reflect the location of poly-A sequences in the genome, or it may correspond to regions with appropriate chromatin structure for endonuclease binding, or it may be a specific adaptation to direct integration to sites where the element is likely to be expressed (i.e. close to promoter/enhancer sequences), while minimising disruption to the host genome.

Some interesting aspects of Zorro3 retrotransposition were also uncovered by the study of elements carrying specific mutations. For example, the finding that Zorro3 elements with nonsense or frameshift mutations in ORF2 can still give rise to retrotransposition events at a detectable frequency suggests either that a low level of nonsense suppression or frameshifting is occurring to allow the occasional translation of ORF2, or, perhaps more likely, that a lack of ORF2 translation can be complemented *in trans *at a low frequency by ORF2 protein produced by endogenous Zorro3 insertions. In support of this possibility, previous work has shown that the endogenous Zorro3 elements are expressed at a readily detectable level [42] and so may be capable of supplying *trans*-acting proteins. If *trans*-activation is occurring then it is interesting to consider why RNA derived from the Zorro3 element carrying the large internal deletion is apparently unable to be *trans*-complemented. One possible explanation is that the extensive deletion in this mutant has removed a sequence which is strictly *cis*-acting. The most obvious difference in the mutations borne by these elements is that in the internal deletion mutant both ORF1 and ORF2 are likely inactivated, whereas only ORF2 is inactivated in the other two. It may be that the product of ORF2 can act *in trans *at some frequency but the product of ORF1 is strictly *cis*-acting.

The results for the mutant in which the first ATG codon in ORF2 is changed to ACG, which show that retrotransposition is reduced to a similar level as that for the ORF2 nonsense and frameshift mutants, suggest that this ATG codon is required for proper ORF2 translation. This is of particular interest as the mechanism by which ORF2 is translated in Zorro3 and other TP elements with non-overlapping ORFs, such as mammalian L1, is not known at present, although mechanisms such as internal initiation or reinitiation have been suggested [57-59]. The results for Zorro3 appear to contrast with those from mammalian L1 elements in that whereas changing the first ATG of ORF2 in L1 to a nonsense codon severely reduced the frequency of retrotransposition, changing it to any other sense codon still allowed for high levels of retrotransposition [59].

The Zorro3 retrotransposition assay system has a number of features which could be improved. In particular, the variability in retrotransposition frequency seen among independent transformants with identical constructs is quite high, and appears to be higher than might be expected simply due to stochastic variation. The source of the variability is currently uncertain, but could arise due to the transforming plasmids integrating into the genome in different copy numbers resulting in different expression levels and therefore different retrotransposition frequencies. If this is the case then the variability might be reduced by expressing the element from an episomal (non-integrative) vector. Unfortunately, the currently available episomal vectors for *C. albicans *[60,61] replicate at low copy numbers, are very unstable, and are prone to randomly integrating into the host genome at a high frequency anyway. These would probably add to the variation rather than reduce it. Another feature of the current assay that might be improved is the Zorro3 retrotransposition frequency, which is quite low in comparison to that of L1 – about 4.5 events per million cells for wild-type Zorro3 compared to as many as 1000 or more per million cells for wild-type L1s [14] (and even higher for extensively modified synthetic L1s [33]). Future attempts to optimise the Zorro3 system may result in increased retrotransposition frequencies.

One possible way of improving the Zorro3 system would be to transfer it to *S. cerevisiae*. However, the successful expression of *C. albicans *genes in *S. cerevisiae *is often not a trivial matter, due to the nonstandard genetic code used by *C. albicans *(in which CUG codons are translated as serine rather than leucine [62]), which may require that all such codons in a *C. albicans *gene are converted to other serine codons prior to expression in *S. cerevisiae*. If, however, the Zorro3 system could be successsfully transferred to *S. cerevisiae*, then this would allow the many genetic tools developed for this species to be employed in the analysis of the retrotransposon. For instance, the contribution of various host genes to the retrotransposition process could be studied by comparing the frequency of retrotransposition in each strain of the *S. cerevisiae *knock-out collection [63]. The potential of the Zorro3 system to function in *S. cerevisiae *is the subject of ongoing research.

## Conclusion

We have developed a yeast model system for studying target-primed retrotransposition. Similar to the way in which the study of specific yeast LTR retrotransposons, such as Ty1 and Ty3, has made an enormously valuable contribution to our general understanding of LTR elements, we hope that this yeast Zorro3 system will make a valuable contribution to our general understanding of target-primed retrotransposons.

## Methods

### Strains and culture conditions

*Candida albicans *strains used were CAI4 [51], a ura3- derivative of the genome sequencing project strain SC5314 [43], and ATCC10261 [64]. *Candida dubliniensis *strains used were CD36, CD41 [65] and CD43 [66]. Strains were grown on YPD medium (1% yeast extract, 2% peptone, 2% glucose) or YNB (0.67% yeast nitrogen base, 1% glucose). When necessary YNB was supplemented with uridine (160 mg/l).

### Standard yeast methods

*C. albicans *DNA isolations, Southern blotting and transformations were as described previously [44]. CAI4 cells were transformed with 3–4 μg of vector linearised with either *Pin *AI or its isoschizomer *Age *I. For selection of *C. albicans *transformants, mycophenolic acid (MPA; Invitrogen), dissolved in absolute ethanol, was added to a final concentration of 5 μg/ml).

### DNA manipulations

Recombinant DNA manipulations were performed using standard techniques [67]. Standard Polymerase Chain Reactions (PCRs) were performed on a Eppendorf Mastercycler gradient instrument, using Expand Hi Fi or Expand Long Template PCR systems (Roche). Oligonucleotides were from Proligo. Sequences of oligos used for plasmid constructions and RT-PCR are available in Additional file 2. Sequences of oligos used for amplification of the ends of specific Zorro3 retrotransposition events are available from the authors on request. Sequencing was performed on a ABI3730 instrument at the Allan Wilson Centre [68].

### RNA isolation and RT-PCR

*C. albicans *RNA was isolated essentially by the method of Schmitt et al. [69] except that cells were grown in YNB+uridine medium. Contaminating DNA was destroyed by treatment with recombinanat, RNase-free DNase I (Roche) as recommended by the manufacturer. Each RNA sample was then separately reverse transcribed with primers ARNAR2 (actin) or ZRNAR2 (Zorro3) using Transcriptor Reverse Transcriptase (Roche) according to the manufacturer's instructions. Initial RT-PCRs were performed as for the standard PCRs above. Quantitative PCRs were performed using a Roche LightCycler 2.0 instrument and LightCycler FastStart DNA MasterPLUS SYBR Green I reaction mix in 20 μl capillaries. *ACT1 *cDNAs were amplified using primers ARNAF2 and ARNAR1. Marked Zorro3 cDNAs were amplified using primers URNAR2 and ZRNAR1. The relative amount of Zorro3 RNA in each sample was normalized to the amount of *ACT1 *RNA in the same sample.

### Sequence data

Sequence data for *Candida albicans *was obtained from the Stanford Genome Technology Center website [70]. Sequencing of *Candida albicans *was accomplished with the support of the NIDR and the Burroughs Wellcome Fund. Data analysed in this report was from Assembly 19 [52]. *Candida dubliniensis *sequence data was obtained from the Sanger Institute [73].

### Plasmid constructions

The Zorro3 retrotransposition vector was made by the sequential assembly of PCR-derived sequences into pUC19-based plasmids as described below. The complete structures of the plasmids used for retrotransposition assays are also described in the Results section. The structures of all clones, and junctions produced by subcloning steps, were verified by sequencing.

The retrotransposition assay plasmid pPZ3TA was constructed as follows. First, a region of the SC5314 genome containing the last 144 bp of the 3' UTR of a full-length Zorro3 element, the poly-A tail (20 consecutive A residues) and 151 bp of 3' flanking sequence was amplified using primers RAZ3F2 and RAZ3R2 (corresponding to bases 7097–7410 of contig19-10054). The PCR product was cut with *Sal *I and *Sph *I and cloned into *Sal *I/*Sph *I-cut pUC19 to give plasmid pZ3.31A. A second PCR product was generated using primers RAZ3F1 and RAZ3R1, corresponding to Zorro3 from a unique central *Xba *I site to the point in the 3' UTR immediately upstream of that included in pZ3.31A. This PCR product was cut with *Asp *718I and *Sal *I and cloned into *Asp *718I/*Sal *I-cut pZ3.31A to give plasmid pZ3XS3.1A. This plasmid contains the entire back end of Zorro3 and 151 bp of 3' flanking sequence and has a unique *Sal *I site inserted into the 3' UTR. Next, a PCR product containing the *C. albicans URA3 *gene disrupted by an antisence intron was generated from plasmid pRUIA [44] using primers U3F1 and U3R1. The PCR products were cut with *Xho *I and cloned into the *Sal *I site of pZ3XS3.1A to give plasmid pZ3UIA3A. In this plasmid the *URA3 *gene is orientated such that the *URA3 *ORF and the Zorro3 ORF are on opposite strands. Next, the front half of Zorro3, including 20 consecutive A residues at the 5' end and extending as far as the unique central *Xba *I site, was amplified by PCR using primers RAZ3F3 and RAZ3R4. The PCR product was cut with *Sac *I and *Xba *I and cloned into *Sac *I/*Xba *I-cut pK19 (a plasmid similar to pUC19 but which confers kanamycin resistance instead of ampicillin resistance) to give plasmid pZ3.51B. The insert of pZ3.51B was then excised as a *Sac *I-*Xba *I fragment and cloned into pZ3UIA3A to give plasmid pZ3AM1A.

The terminator region of the *C. albicans ACT1 *gene [71] was amplified by PCR from SC5314 genomic DNA using primers TAF1 and TAR1. The PCR product was cut with *Sal *I and *Sph *I and cloned into *Xho *I/*Sph *I-cut pZ3AM1A to give plasmid pZ3TA.

The *C. albicans RP10 *gene [47] was amplified using primers R10F1 and R10R1. The PCR product was cut with *Bam *HI and *Pst *I and cloned into *Bam *HI/*Pst *I-cut pK19 to give plasmid pRP10.1A.

An allele of the *C. albicans IMH3 *gene [45,46] that confers resistance to high levels of the antibiotic mycophenolic acid (MPA) was amplified from plasmid pSFI1A [ref. [72]; kindly provided by Dr Joachim Morschhauser and Dr Gerwald Kohler], using primers I3F1 and I3R1. The PCR product was cut with *Eco *RI and *Bam *HI and cloned into *Eco *RI/*Bam *HI-cut pRP10.1A to give plasmid pR10I31C.

The *IMH3*/*RP10 *genes of pR10I31C were cut out with *Sal *I and cloned into the *Xho *I site of pZ3TA to give plasmid pZTMAA.

The promoter region of the *C. albicans ACT1 *gene was amplified by PCR from SC5314 genomic DNA using primers ACTF1 and ACTR1. The PCR product was cut with *Xba *I and cloned into the *Spe *I site immediately upstream of the 5' end of the Zorro3 element in pZTMAA to give plasmid pPZ3TA, the final plasmid on which the retrotransposition assay is based.

A derivative of pPZ3TA containing a deletion within the Zorro3 sequence was constructed by cleaving the plasmid with *Pst *I and self-ligating to produce plasmid pPZ3TAΔPA. In this plasmid, the Zorro3 element lacks all sequences between a *Pst *I site near the middle of ORF1 and a *Pst *I site near the middle of ORF2, but is otherwise identical to pPZ3TA.

Several specific mutants of the tagged Zorro3 element were created by first replacing a 410 bp fragment of pPZ3TA between a unique *Bfu *AI cleavage site 6 bp upstream of the ORF1 stop codon and a unique *Nsi *I site 220 bp downstream of the start of ORF2 with a *Bfu *AI-*Nsi *I fragment containing a chloramphenicol resistance gene, amplified using primers CATF1 and CATR1 from a pBluescript derivative (pBCTim1A) containing a modified cloning cassette (unpublished), to produce plasmid pZ3ΔBN.CAT1A. Six different PCR products of the Zorro3 *Bfu *AI-*Nsi *I fragment, each containing either the wild-type sequence or one of five specific mutations, were then used to replace the *Bfu *AI-*Nsi *I fragment of pZ3ΔBN.CAT1A, giving plasmids pZ3BN1 (wild-type) and pZ3BN2 to pZ3BN6 (five different mutants). The wild-type sequence was amplified using primers MZ3F1 and MZ3R1. The pZ3BN2 mutant (containing a stop codon in ORF2) was produced using primers MZ3F1 and MZ3R4. The pZ3BN3 mutant (containing a frameshift in ORF2) was produced using primers MZ3F1 and MZ3R5. The pZ3BN4 mutant (in which the first ATG codon in ORF2 was changed to ACG) was produced by combining PCR products made using primers MZ3F1+MZ3R2 and MZ3F2+MZ3R1. The pZ3BN5 mutant (in which an A-rich sequence in the interORF region was replaced with an *Asp *718I site) was produced by combining PCR products made using primers MZ3F1+MZ3R3 and MZ3F3+MZ3R1. The pZ3BN6 mutant (in which a conserved Asn codon within the EN coding region was replaced with an Ala codon (N17A)) was produced by combining PCR products made using primers MZ3F1+MZ3R6 and MZ3F4+MZ3R1.

### Zorro3 retrotransposition assay

CAI4 transformants containing marked copies of Zorro3 were grown at 37°C in liquid YNB medium supplemented with uridine and MPA for two days. Multiple aliquots (250 μl each) were then plated onto sets of YNB plates (usually eight plates per transformant) and incubated at the desired temperature. Counts of colonies were then made daily for several weeks beginning after three or four days. Estimates of the numbers of cells on the retrotransposition assay plates were made by plating 250 μl aliquots of serial 10-fold dilutions of the original liquid culture onto YNB plates supplemented with uridine. Plates were incubated at the same temperature as the assay plates. Colonies were counted after 2–4 days. Note that the liquid cultures were grown at 37°C because it was found that retrotransposition did not occur at this temperature, thus ensuring that colonies that arose on the plates were due to independent retrotransposition events, rather than potentially being clones of a single event that occurred within the culture.

## Accession numbers

Accession numbers are as follows: Full-length Zorro3, GenBank:AF254443; Zorro3 expression vector pPZ3TA, GenBank:EF667963; 5' end of Zorro3 insertion A from ATCC10261, GenBank:DQ239689; 5' end of Zorro3 insertion B from ATCC10261, GenBank:DQ239690.

## Abbreviations

EN, Endonuclease; LTR, Long Terminal Repeat; MPA, Mycophenolic Acid; ORF, Open Reading Frame; PCR, Polymerase Chain Reaction; RT, Reverse Transcriptase; TP, Target-Primed; UTR, Untranslated Region; ZF, Zinc Finger.

## Authors' contributions

TG designed the experiments and performed most of the experimental work, analysed the data and wrote the manuscript. RP conceived the study and participated in the design of experiments, the analysis of the data and the writing of the manuscript. JB collected and analysed many of the Zorro3 retrotransposition events. All authors read and approved the final manuscript.

## Supplementary Material

Additional file 1 Zorro3 insertion sites in relation to neighbouring ORFs. A Microsoft Word table of Zorro3 insertion site data. Click here for file

Additional file 2 Oligonucleotides used for plasmid constructions and RT-PCR. A Microsoft Word table of oligonucleotide sequences. Click here for file
