# Supplementary material for: A yeast model for target-primed (non-LTR) retrotransposition
Source: BMC Genomics. 2007 Aug 3;8:263. doi: 10.1186/1471-2164-8-263 (PMC1965478; doi:10.1186/1471-2164-8-263)
Supplement: Additional file 1 — Zorro3 insertion sites in relation to neighbouring ORFs. A Microsoft Word table of Zorro3 insertion site data. [file 1471-2164-8-263-S1.doc]

Supplementary Table S1. Zorro3 insertion sites in relation to neighbouring ORFs.

| Insert no. | Assembly 19 contig^A^ | Chromo-  some | Distance to upstream ORF (end)^B^ | Distance to downstream ORF (end)^B^ |
| --- | --- | --- | --- | --- |
| 12.1 | x0254  (94830 F) | 3 | 420 (5′) | 1530 (5′) |
| 12.2 | x0257  (1660 F) | 2^C^ | 130 (5′) | 160 (5′) |
| 12.4 | 10119  (254650 F) | 2 | 200 (5′) | 250 (5′) |
| 22.1 | x0163  (134670 R) | 1 | 140 (5′) | 320 (5′) |
| 13.3 | x0234  (49865 R) | 2 | 230 (5′) | 170 (3′) |
| 13.4 | 10231  (18975 F) | 4 | 230 (5′) | 380 (5′) |
| 13.5 | x0143  (96420 F) | 2 | 150 (3′) | 70 (5′) |
| 13.6 | x0163  (203035 F) | 1 | 900 (5′) | 1410 (5′) |
| 13.7 | x0119  (244340 F) | 2 | 120 (5′) | 100 (5′) |
| 13.11 | 10063  (36770 F) | R | 1620 (5′) | 3710 (3′) |
| 13.13 | x0216  (94130 R) | 1 | 1320 (5′) | 580 (5′) |
| 13.14 | 10212  (133650 R) | 4 | 610 (5′) | 340 (3′) |
| 13.15 | x0196  (22910 F) | 2 | 120 (5′) | 4420 (5′) |
| 13.17 | x0166  (27780 R) | 4 | 120 (5′) | 100 (3′) |
| 13.18 | x0163  (15740 F) | 1 | 1380 (5′) | 2560 (3′) |
| 14.1 | x0087  (72350 R) | 1 | 1350 (5′) | 710 (3′) |
| 14.4 | 20236  (26790 R) | 3 | 630 (5′) | 2260 (3′) |
| 14.5 | x0218  (144180 R) | 1 | 280 (5′) | 1100 (3′) |
| 14.7 | x0188  (25725 F) | R | 760 (5′) | 2040 (5′) |
| 14.11 | x0139  (200180 F) | 2 | 100 (5′) | 40 (5′) |
| 14.13 | 10020  (2820 R) | 1 | 380 (3′) | 1010 (5′) |
| 14.15 | x0087  (72350 R) | 1 | 1350 (5′) | 710 (3′) |
| 14.16 | 2500  (72900 R) | 3 | 210 (5′) | 920 (5′) |
| 14.17 | x0073  (13260 R) | 1 | 390 (3′) | 320 (5′) |
| 14.18 | x0163  (134670 R) | 1 | 140 (5′) | 320 (5′) |
| 14.19 | 2506  (28525 R) | 7 | 1310 (3′) | 3570 (3′) |
| 14.20 | 20161  (92640 R) | R | 3620 (5′) | 730 (5′) |
| 14.21 | 20216  (97180 F) | 1 | 460 (3′) | 1810 (5′) |
| 14.22 | 10119  (254650 F) | 2 | 200 (5′) | 240 (5′) |
| 14.24 | 20155  (47610 R) | 5 | 3450 (5′) | 180 (5′) |

^A^ Assembly 19 contigs generally come in pairs of similar sequence representing portions of putative homologous chromosome pairs. Members of the pairs are distinguished by either a 1 or 2 at the start of the contig number, eg. 10254 and 20254. In the cases where the Zorro3 insertion site can be assigned to a particular homolog the number of that contig is shown. In cases where the insertion could lie on either of the homologs the first digit of the contig number is replaced by an x. The positions of the insertions within the contigs and their orientations, forward (F) or reverse (R), are shown in parentheses.

^B^ The distance in base pairs to the nearest upstream or downstream ORF is shown and the orientation of that ORF (i.e. whether the 5′ or the 3′ end lies closest to the Zorro3 insertion site) is indicated in parentheses. Insertion site data refers to the 3′ end of the Zorro3 element in cases where the 5′ end has apparently recombined with an endogenous insertion. ORF data was derived from the Assembly 19 of the genome sequence.

^C^ Insertion site corresponds to chromosome 2 in *C. albicans* strain WO-1. The chromosomal origin of contigs 19-10257 and 19-20257 in strain SC5314 is not known due to conflicting data.
