# Supplementary material for: A yeast model for target-primed (non-LTR) retrotransposition
Source: BMC Genomics. 2007 Aug 3;8:263. doi: 10.1186/1471-2164-8-263 (PMC1965478; doi:10.1186/1471-2164-8-263)
Supplement: Additional file 2 — Oligonucleotides used for plasmid constructions and RT-PCR. A Microsoft Word table of oligonucleotide sequences. [file 1471-2164-8-263-S2.doc]

Supplementary Table S2. Oligonucleotides used for plasmid constructions and RT-PCR

OligoSequence (5’–3’)ACTF1CGTTCTCTAGAGCTCGATAGAGCTATTAAGATCACCACTR1CGTTGTCTAGATTTGAATGATTATATTTTTTTAATATTAATATCGAGARNAF1TATAATCATTCAAAATGGACGGTGARNAF2ATCATTCAAAATGGACGGTGAAGAAARNAR1TGGATTCATTGGAGCTTCGGTCAACARNAR2GGAAACGTAGAAAGCTGGAACACATF1TCCAGGGCTAAACCTGCCACCGAACCGAATAAATACCTGTGACGCATR1GACTGATGCATGCGGCCGCGATCATATCGTCAATTATTACCTCCACI3F1CCTGGGATCCTGTTTATGATACTAAGGTAAAGTGGI3R1CCTGGAATTCGTCGACAGAACTCAGTATATCTTCATCMZ3F1GAGACACTAGTGGATCCAGGGCTAAACCTGCMZ3F2GAGAAATGAATCCTATATTAACAGMZ3F3GACTGGGTACCTTCATATTCATACATACATACTTACACCMZ3F4GGCTCCAAAGCAATTGGCAGCCATCAATCCMZ3R1GACTGGAATTCATGCATTCTAAGCTGTGCATTGTGMZ3R2AATATAGGATTCATTTCTCTTCGTTCACAGTAATTAAATAATGGMZ3R3GACTGGGTACCGTATTATAAATAACGGGAAAATCMZ3R4GACTGGAATTCATGCATTCAAAGCTGTGCATTGTGTTGMZ3R5GACTGGAATTCATGCATTTCTAAGCTGTGCATTGTGTTGMZ3R6GCTGCCAATTGCTTTGGAGCCTATTGTTAAACRAZ3F1CCTTCGGTACCAATTCTAGAAGAGTTTAAGCAAATCTCGRAZ3F2CCTTCGTCGACGATATTCTCACTGAGTATTTCTTTCCRAZ3F3CCTTCGAGCTCTTACTAGTAAAAAAAAAAAAAAAAAAAATTATTATGACTACGAACGGTACRAZ3R1CCATCGTCGACTTAAAAACCATCGTGTATGATATGTGGRAZ3R2CCATCGCATGCGGCCGCTCGAGTGCATTACTAACACGACGAGGRAZ3R4GCTTAAACTCTTCTAGAATTCGGCAGTTTCGR10F1CGTCGGATCCTGTGCGTTGTTATCCTGCACACR10R1CGTCCTGCAGGTCGACATCACTACAACATCTCGTATTCACTAF1CTGGAGTCGACTAAGAGTGAAATTCTGGAAATCTGGTAR1CTGGAGCATGCTCGAGATTTTATGATGGAATGAATGGGU3F1CGTCATGCATCTCGAGTTCTTCAATGATGATTTCAACCATTCU3R1CGTCATGCATCTCGAGCCTTCACATTTATAATTGGCCAGTCURNAR1GGTTTTCTTCAGTTCCATTAATCGAURNAR2CTCACTATAGGTCTTAGTGTTGACZRNAR1TTTGAGGCCATTTTCATGGAAAGZRNAR2TATATTTAGTCCTTATATACAGGT
